# FI: The Fecobiome Initiative

**DOI:** 10.1089/fpd.2021.0082

**Published:** 2022-07-11

**Authors:** Panagiotis Sapountzis, Serafino Teseo, Saria Otani, Frank Møller Aarestrup, Evelyne Forano, Garett Suen, George Tsiamis, Bradd Haley, Jo Ann Van Kessel, Sharon A. Huws

**Affiliations:** ^1^ Université Clermont Auvergne, INRAE, UMR 0454 MEDIS, Clermont-Ferrand, France.; ^2^ School of Biological Sciences, Nanyang Technological University, Singapore, Singapore.; ^3^ National Food Institute, Technical University of Denmark, Kongens Lyngby, Denmark.; ^4^ Department of Bacteriology, University of Wisconsin-Madison, Madison, Wisconsin, USA.; ^5^ Lab of Systems Microbiology and Applied Genomics, University of Patras, Agrinio, Greece.; ^6^ Environmental Microbial and Food Safety Laboratory, Beltsville Agricultural Research Center, Agricultural Research Service, United States Department of Agriculture, Beltsville, Maryland, USA.; ^7^ School of Biological Sciences, Institute for Global Food Security, Queens University Belfast (QUB), Belfast, United Kingdom.

**Keywords:** research initiative, gastrointestinal tract, microbiota, zoonoses

## Abstract

Animal husbandry has been key to the sustainability of human societies for millennia. Livestock animals, such as cattle, convert plants to protein biomass due to a compartmentalized gastrointestinal tract (GIT) and the complementary contributions of a diverse GIT microbiota, thereby providing humans with meat and dairy products. Research on cattle gut microbial symbionts has mainly focused on the rumen (which is the primary fermentation compartment) and there is a paucity of functional insight on the intestinal (distal end) microbiota, where most foodborne zoonotic bacteria reside. Here, we present the Fecobiome Initiative (or FI), an international effort that aims at facilitating collaboration on research projects related to the intestinal microbiota, disseminating research results, and increasing public availability of resources. By doing so, the FI can help mitigate foodborne and animal pathogens that threaten livestock and human health, reduce the emergence and spread of antimicrobial resistance in cattle and their proximate environment, and potentially improve the welfare and nutrition of animals. We invite all researchers interested in this type of research to join the FI through our website: www.fecobiome.com

## Background

Cattle have been our domesticated associates since the dawn of agricultural societies, ca 10,000 years ago. With the complementary contributions of a highly specialized gut microbiota, they efficiently convert plant material into protein mass, utilizing dietary components that cannot be digested by monogastrics. This ability has made them our valuable partners since their domestication, as they provide us with a stable supply of protein in the form of meat and dairy products.

**FIG. 1.**
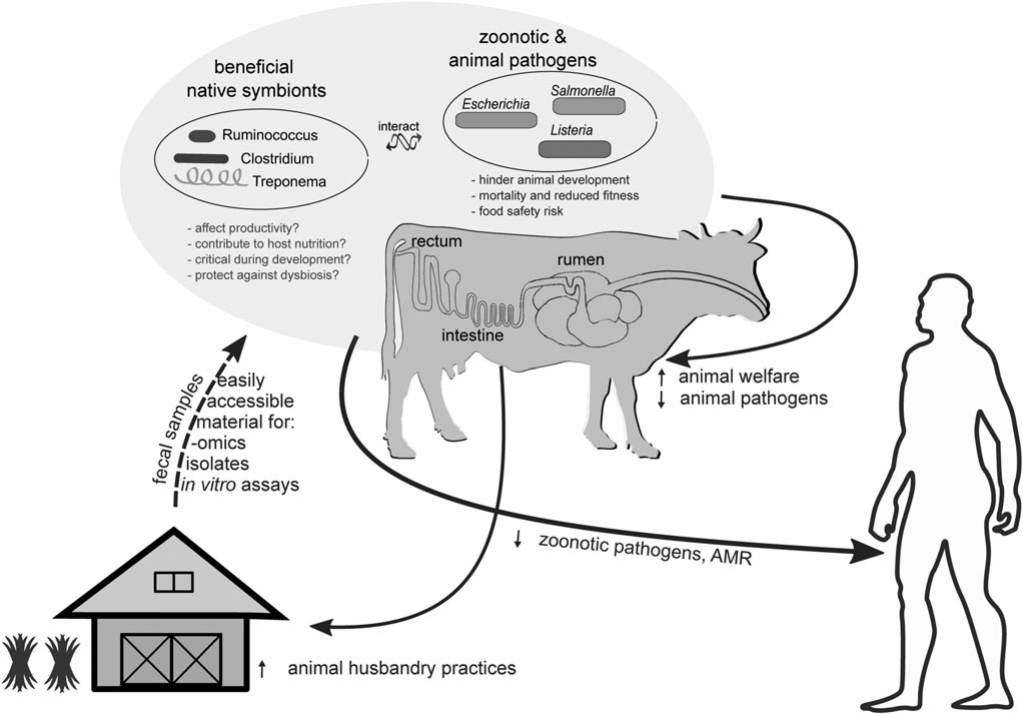
Overview of the FI vision and goals, the relevant research areas, and how successful implementation could improve animal health and nutrition and reduce the zoonotic and AMR exposure risk to humans. Black arrows show benefits. On top (highlighted with a light blue background), the two main microbial groups of interest (separated based on whether they are beneficial mutualists or animal/zoonotic pathogens) and their implications to potential human exposure and animal husbandry practices are presented. AMR, antimicrobial resistance; FI, Fecobiome Initiative.

As expected, the microbial community residing in the gastrointestinal tract (GIT) of cattle has been the focus of research efforts for more than half a century. The rumen is the primary fermentation site (Fig. 1) where a complex microbial community, composed of archaea, bacteria, protozoa, and fungi, is responsible for the degradation and fermentation of dietary components and the provision of digested nutrients to the host (Koike and Kobayashi, 2009; Dodd *et al.*, 2011; Comtet-Marre *et al.*, 2017; Huws *et al.*, 2018; Seshadri *et al.*, 2018; Stewart *et al.*, 2018; Kameshwar *et al.*, 2019; Mizrahi *et al.*, 2021).

When partially digested feed and microbial biomass pass through the rumen, digestion continues in the other GIT compartments, until the contents reach the large intestine (Fig. 1), the last section of the GIT. Here, water and nutrients are absorbed (Moran, 2005; Bergmann, 2017), whereas a prominent microbial biomass (second only to that of the foregut) further ferments the remaining dietary components.

The large intestine is of crucial interest not only because of the metabolic processes in which its resident microbiota engage, but also because it is the primary colonization site of zoonotic and animal pathogens. Cattle, for example, are the primary reservoir of the Shiga toxin-producing *Escherichia coli* (STEC). Because STEC pose no threat to an adult host, they do not trigger any host defenses; they exploit the commensal microbiota to transit through the rumen and subsequently colonize the rectal mucosa of the large intestine (Naylor *et al.*, 2003; Sapountzis *et al.*, 2020). Several 16S rRNA amplicon sequencing studies have suggested that members of the cattle rectal microbiota may be able to exclude zoonotic STEC based on mutual exclusion patterns between the rectal microbial community and STEC (Xu *et al.*, 2014; Zaheer *et al.*, 2017; Stenkamp-Strahm *et al.*, 2018; Wang *et al.*, 2018). Other *E. coli* zoonotic pathotypes include the enterotoxigenic *E. coli*, which colonize the small intestine (Fig. 1) and can be detected in animal feces (Nagy and Fekete, 1999).

Similar to *Escherichia*, *Salmonella* pathotypes infect cattle through the fecal–oral route (Holschbach and Peek, 2018) and colonize the rectal mucosa (Palmer *et al.*, 1985); the establishment of several pathotypes has been suggested to be prevented by a healthy cattle GIT microbiota (Holschbach and Peek, 2018), although experimental evidence is lacking. Other known zoonotics of ruminants include *Clostridium perfringens* (Fohler *et al.*, 2016), *Listeria monocytogenes*, and *Campylobacter* species (Hannon *et al.*, 2009).

In addition to zoonotic pathogens, which are often but not always, harmless hitchhikers of the bovine host, the large intestine is also an Achilles heel for young bovines, which are susceptible to bacterial infections. Early in life, the epimural microbial communities of the intestine are critical for the development and homeostasis of the GIT immune system (Malmuthuge and Guan, 2017; Huws *et al.*, 2018), whereas certain members, such as lactic acid bacteria, which are dominant in very young calves (Alipour *et al.*, 2018), appear to protect against enteric infections and gut dysbiosis (Malmuthuge and Guan, 2017; Morgavi *et al.*, 2020).

The rapidly developing field of genomics has played an important role in deciphering functions of the symbiotic GIT microbes of animals, including humans, as well as the monitoring of antimicrobial resistance (AMR) and virulence genes. Characterization of the genetic machinery of gut microbes has improved our understanding of: (i) mutualistic (typically nutritional) services gut microbes provide to their hosts; (ii) microbial interactions; and (iii) proximate and evolutionary mechanisms shaping genomes, including factors that facilitate the development of pathogenic traits that can exploit the host and/or associated microbial communities.

Besides the nutritional aspects that may apply to almost all animals (Bourtzis and Miller, 2003; Matthews *et al.*, 2019; Gardiner *et al.*, 2020; Jahnes and Sabree, 2020), the interplay between microbiota and host has also been proven to impact all aspects of the host's health, development, hormone regulation, and behavior, as almost every physiological process is potentially affected by the microbes residing in the GIT (Hara *et al.*, 2013; Kamada *et al.*, 2013; Kanji *et al.*, 2017; Malmuthuge and Guan, 2017; Butt and Volkoff, 2019; Teseo *et al.*, 2019; Liberti and Engel, 2020; Sapountzis *et al.*, 2020).

For the past 10 years, the use of metagenomics and next-generation sequencing studies has led to important insights in all the earlier mentioned research areas, allowing researchers to: (i) take an integrated approach to the characterization of microbial gene inventories (shotgun metagenomics) and the reconstruction of metagenome-assembled genomes of uncultured symbionts, also known as “dark matter” (Bernard *et al.*, 2018); (ii) perform quantitative comparisons of the diversity and structure of microbial communities (biomarkers such as 16S, 18S, and ITS).

In cattle, genomic approaches (both culture and nonculture-based) have mostly focused on the rumen (Pitta *et al.*, 2016; Seshadri *et al.*, 2018; Stewart *et al.*, 2018; Bickhart *et al.*, 2019). Despite the clear role of the large intestine in many aspects of health, nutrition, and the spread of zoonotic pathogens and AMR, the knowledge of its microbiota is mainly limited to:

(i) descriptive 16S/18S rRNA targeted sequencing (Holman and Gzyl, 2019),
(ii) single bacterial species (i.e., *Escherichia* and *Salmonella*) and their antagonists that pose a threat to the cattle host or human consumers (Wang *et al.*, 2017; Holschbach and Peek, 2018), and
(iii) the occurrence and distribution of AMR genes in the feces (Noelle Noyes *et al.*, 2016a, b; Doster *et al.*, 2018; Zaheer et al., 2018, 2019; Keijser *et al.*, 2019; Liu *et al.*, 2019; Rovira *et al.*, 2019; Weinroth *et al.*, 2019; Gaeta *et al.*, 2020; Haley *et al.*, 2020; Lim *et al.*, 2020; Salaheen *et al.*, 2020; Duarte *et al.*, 2021) (see also https://www.fecobiome.com/resources/data/).

As of now, very few functional studies or shotgun metagenomic studies have taken an integrated approach to explore the genomic potential of the beneficial microbial residents, the characterization of animal pathotypes or zoonotic hitchhikers and their interactions with the host or each other, the ecological factors impacting their biology, or the potential relationship between microbiota characteristics and the spread of AMR and virulence factors.

We believe it is timely to establish an initiative that can identify common ground between these types of projects, help exploit the full potential of existing sequencing data and other resources, and provide a platform for the emergence of ideas that can generate novel functional interdisciplinary projects. Examples of such projects include the genomic and functional deciphering of the microbial diversity of the large intestine, which can provide the cornerstone for all kinds of follow-up transcriptional, chemical, or metabolomic studies and a global effort to collect microbial isolates representing members from all genera that can be found in cattle GIT.

By helping the emergence of such projects (via experimental and intellectual collaboration), which, in turn, can lead to a better understanding of the global microbial diversity of the GIT microbiome, we are confident that the Fecobiome Initiative (FI) can ultimately limit zoonotic pathogens from entering our food chain, better control animal pathogens that hinder the animal development and productivity, reduce the emergence of AMR, optimize the use of antibiotics in animal husbandry, and potentially maximize the nutritional benefits provided by the microbial community to the cattle host.

## FI Vision

We hereby propose the formation of the FI (similar to the Greek letter Φ; also known as the golden ratio in mathematics), an international effort that will promote scientific cooperation, dissemination of research findings, and data transparency to facilitate the advancement of research related to microbial communities primarily of the large intestine of cattle, but also related to the rest of the GIT. The vision of the FI is to offer viable solutions in cattle husbandry that will help reduce the zoonotic and AMR risk, animal pathogens, and reduce or optimize the use of antibiotics, with the goal of improving animal welfare while enhancing meat and dairy production.

## FI Mission

Through a dedicated website (www.fecobiome.com) that will serve as a coordination and dissemination agent, the FI will:

(i) bring together and coordinate collaboration among members of the scientific community (e.g., by organizing workshops to align strategies for funding applications and joint proposals) who are interested in the study of microbes residing in the GIT (primarily the large intestine) of cattle,
(ii) disseminate knowledge by using language and material targeting not only scientists (working on cattle microbiota-related research) but also nonspecialists (e.g., again by organizing events like webinars or workshops for training purposes) and stakeholders involved in animal husbandry, and thus help bridge the gap between the scientific community and nonsubject matter specialists,
(iii) increase public availability of protocols, relevant material (e.g., primers, probes, antibodies), and isolated microbial strains; and by making data (genomic, metagenomic or other) more accessible to the public (i.e., by contributing whenever possible with supplementary metadata and a built-in interface that will allow easier search and recovery of genomic data).

## Why Feces?

The organizers of the FI are interested in the study of gut microbes residing primarily in the last compartment of the cattle GIT, the large intestine. To achieve this, the FI will focus on samples originating from fresh feces as: (i) they present a reliable representation of the microbial community at the distal end of the GIT, and (ii) they do not need special permits for handling of animals in commercial or other farms. In addition, feces can also contain microbial isolates from representatives of other parts of the GIT such as the small intestine and the rumen, which will be valuable for research related to these parts of the GIT (see also the RMG network; https://rmgnetwork.org/).

Fresh feces is an excellent sample for: (i) the isolation of cultivable microbes; (ii) isolation and characterization of nucleotide or protein material for -omics approaches, diagnostic polymerase chain reaction, or other molecular and biochemical methods; (iii) chemical analyses for determining nondigested feed components; and (iv) *in vivo* and *in vitro* experimentation. We would note that, despite our interest in the microbial communities of fecal samples, FI does not argue against the use of material from the GIT of animals that has been collected via direct rectal sampling (e.g., rectal swabs) or necropsies (i.e., GIT parts from slaughtered calves) as this material will remain invaluable for other research questions.

## FI Goals

The FI has the potential to benefit research in the areas of ecology, evolution, epidemiology, microbiology, human health, and medicine and veterinary medicine, and it may lead to solutions for the improvement of animal husbandry practices. Some examples include:

- Characterization of the distal end microbiome will allow us to formulate strong hypotheses about the nature of the relationship between members of the microbial community and the cattle host, the intertwined dynamics among the community members (cooperative or competitive interactions), and the functional complementarity or redundancy across compartments (e.g., when examined in comparison with similar available datasets from the rumen; Hungate dataset). Insights gleaned from such studies can significantly improve our understanding of the homeostasis and functioning of the GIT microbial community.
- Characterizing the microbial variability on a global scale will allow us to link the variability to host and environmental factors (through correlations of big data sets) such as climate, geographic location, diets, and farm management practices that may affect microbial establishment and resilience. This will allow us to formulate hypotheses about the key factors that influence the GIT microbial community.
- The Whole Genome Sequencing approaches may reveal patterns of genomic evolution, since comparisons of the isolated bacterial strains with their close relatives can reveal traits that have evolved as a result of the unique ecosystem in the large intestine.

The FI can directly contribute to applied solutions in animal husbandry, including the discovery of novel direct-fed microbials to potentially protect against pathogens during early development, to prevent zoonotic pathogens from entering and establishing themselves in the GIT, to limit the spread of AMR bacteria, and potentially to improve animal nutrition and productivity.

In addition, the FI could lead to improvements in the assessment of zoonotic risks, since currently zoonotic risk and the presence of pathogens have been evaluated by targeting only a handful of known pathogens (mainly *Escherichia* and *Salmonella* genera) and with culture-based approaches. By promoting culture-independent approaches that examine the entire fecal community in parallel with culture-based approaches, we will enhance our ability to identify emerging pathogens or other threats.

## Short-Term and Long-Term Objectives and Benefits to the Community

The FI will identify knowledge gaps and focus on research that will complement existing knowledge regarding the microbial inhabitants of the large intestine.

To achieve this, the FI will prioritize research areas such as:

(i) Characterization of the microbial community of cattle feces by using high-quality/in-depth shotgun metagenomic sequencing to make detailed gene catalogs of the microbial genomic inventory present in the large intestine and identify potential pathogenic and AMR-associated genes.
(ii) Isolation of microbial strains from fecal samples using both taxa-specific and nonspecific approaches and create a representative library of native aerobic and anaerobic isolates.
(iii) Using complementary -omics approaches to assemble evidence-based data as these approaches can link, among other factors, DNA sequences, proteins, and metabolites.
(iv) Large-scale correlations between (meta) genomic data to identify potential factors responsible for the variations between microbial communities (e.g., ecological and geographic datasets, diets, husbandry practices, breed, age, sex, etc.). These correlations require data and isolates from cattle of every breed and age around the globe. We recognize this is an ambitious goal, but we are hopeful that, once the FI is launched, the FI can facilitate data collection, leading to novel results that fuel future projects that allow us to achieve our goals.

We note that the dissemination of data and knowledge is one of the core missions of the FI and toward this end we plan to create a wiki portal (as part of the fecobiome.com website) that will present relevant scientific articles in a simplified and concise view to allow nonsubject matter experts to better connect with the scientific community. Bridging the gap between animal husbandry stakeholders and researchers has the potential to significantly increase the availability of fecal samples from farms, as currently the communication gap between these two parties is often an obstacle.

As (meta)genomic data accumulate, the map of fecal microbial diversity will become more complete, and accomplishing the FI short- and long-term objectives will aid all projects related to the GIT distal end microbiome:

- Microbial isolations and genomic sequencing will provide reference genomes that will serve as templates for meta-genomic, transcriptomic, and proteomic studies and the identification of novel key genes and taxa (e.g., to be used as markers). This includes pathogenic taxa and virulence genes that can be used as biomarkers, as global comparisons will allow for the evaluation of the most relevant threats.
- Complementary *in vitro* and *in vivo* methods applied to microbial isolates can evaluate their primary and secondary metabolic potential and growth dynamics; insight that, when coupled with metagenomic data, can provide a reconstruction of the community metabolic network and potential ecological interactions.
- Characterizing the large intestine microbes will foster synergies with existing networks related to the cattle GIT microbiome (i.e., RMG network) and ongoing projects, as the combined results can provide an overview of the community structure and mechanisms along the GIT and allow for an integrated approach to the study of the GIT.

## Launching the FI

Considering the growing number of shotgun metagenomic fecal data in cattle during the past 3 years (Noelle Noyes *et al.*, 2016a, b; Doster *et al.*, 2018; Zaheer et al., 2018, 2019; Liu *et al.*, 2019; Rovira *et al.*, 2019; Salaheen et al., 2019, 2020; Weinroth *et al.*, 2019; Gaeta *et al.*, 2020; Haley *et al.*, 2020; Lim *et al.*, 2020; Duarte *et al.*, 2021), launching the FI is timely, as its website (fecobiome.com) can serve as a central repository to organize metadata and facilitate meta-analyses to fully exploit the potential of such data.

For this purpose, we have developed a search tool at fecobiome.com (a beta version is already available online at: www.fecobiome.com/resources/) that will allow users to easily locate genomic and other data of interest. Researchers can submit the metadata of their studies through the website (https://www.fecobiome.com/contact/submit-data/). Fecal microbial isolates are already available at the National Research Institute for Agriculture, Food and the Environment (France), the Institute for Global Food Security at Queen's University Belfast (Ireland), and the Danish Technological Institute (Denmark) and the metainformation of these isolates will be shortly available at our website. Similarly, a list of protocols and reagents along with research labs that can provide them will also be made publicly available through www.fecobiome.com.

The FI website will serve as a platform for connection and communication among members (an internet forum is an upcoming feature), where members can exchange ideas related to our initiative. The website administrators will be responsible for regulating the threads and for monthly news updates. An Internet forum can also facilitate communication between members, which will initially take place through the website. However, in the coming years, the FI will actively pursue funding for organizing meetings and workshops where members can interact in person.

We invite all interested parties to join the FI. It will be a dynamic site and, through the continuous feedback of members, the FI will adapt to the needs of fecobiome related research in the coming years.
